# The effects of olibanum on male reproductive system damage in a lipopolysaccharide induced systemic inflammation model in rat

**DOI:** 10.1016/j.heliyon.2024.e36033

**Published:** 2024-08-09

**Authors:** Narjes Jalilvand, Yousef Baghcheghi, Masoumeh Fani, Farimah Beheshti, Alireza Ebrahimzadeh-Bideskan, Narges Marefati, Maryam Moghimian, Mahmoud Hosseini

**Affiliations:** a Psychiatry and Behavioral Sciences Research Center, Mashhad University of Medical Sciences, Mashhad, Iran; b Department of Physiology, School of Medicine, Mashhad University of Medical Sciences, Mashhad, Iran; c Student Research Committee, Jiroft University of Medical Sciences, Jiroft, Iran; d Department of Anatomy, School of Medicine, Gonabad University of Medical Sciences, Gonabad, Iran; e Neuroscience Research Center, Torbat Heydariyeh University of Medical Sciences, Torbat Heydariyeh, Iran; f Department of Physiology, School of Medicine, Torbat Heydariyeh University of Medical Sciences, Torbat Heydariyeh, Iran; g Applied Biomedical Research Center, Mashhad University of Medical Sciences, Mashhad, Iran; h Department of Anatomy and Cell Biology, Faculty of Medicine, Mashhad University of Medical Sciences, Mashhad, Iran; i Department of Physiology and Medical Physics, Faculty of Medicine, Baqiyatallah University of Medical Sciences, Tehran, Iran; j Nursing Research Center, Gonabad University of Medical Sciences, Gonabad, Iran; k Department of Physiology, School of Medicine, Gonabad University of Medical Sciences, Gonabad, Iran; l Neuroscience Research Center, Mashhad University of Medical Sciences, Mashhad, Iran

**Keywords:** Olibanum, Lipopolysaccharide, Germ cell, Apoptosis, Oxidative stress

## Abstract

**Background:**

Lipopolysaccharide (LPS) as a particle of Gram-negative bacteria is a main contributer in the pathogenesis of the male reproductive system infectious. Male infertility due to LPS is reported to be related to overproduction reactive oxygen species. This study aimed to investigate the effects of olibanum on oxidative stress and apoptosis in testes and sperm dysfunction induced by LPS.

**Methods:**

The male (n = 28) rats were allocated in four groups: control, LPS (1 mg/kg, i.p., 14 days), LPS + Olibanum 100 (100 mg/kg, i.p., 14 days), and LPS + Olibanum 200 (200 mg/kg, i.p., 14 days). Germ cell apoptosis was determined by TUNEL assays and computed using the stereological method. Additionally, semen samples of the animals were analyzed for sperm count and morphology. Oxidative stress indicators were also determined.

**Results:**

The count of TUNEL-positive germ cells in LPS-treated rats was more than that in the controls. Treatment of the animals with olibanum significantly attenuated the number of apoptotic cells compared to the LPS group. The sperm count and those with a normal morphology in LPS-treated rats was lower than that in the controls. Administration of olibanum significantly improved the sperms with normal morphology and sperm count. Olibanum treatment also improved superoxide dismutase, catalase, and total thiol in testicular tissue and decreased malondialdehyde.

**Conclusion:**

Administering both doses of olibanum in LPS-treated rats had potentially a therapeutic value in reducing germ cell apoptosis, as well as improving sperm parameters.

## 1 Introduction

More than half of all infertility cases are due to male-related diseases and about one in twenty males are affected by infertility factors [1]. Male infertility is sometimes attributed to inflammatory diseases which arise from infections by bacteria [2]. Lipopolysaccharide (LPS) is a part membrane of Gram-negative bacteria which can cause a systemic inflammation and accompanuied with an oxidative stress condition in the host [3,4]. Therefore, the LPS-induced inflammation rodent models can serve as a valuable way to determine the relationship between oxidative stress, inflammation, and infertility [5]. LPS is well known that activates the immune cells including mast cells and neutrophils [6,7]. LPS injection in animal models also leads to inflammatory response and an overproduction reactive oxygen species (ROS) [3,4,8]. Moreover, previous studies have found that injection of LPS into rats induces the ROS generation in the testes and it has been well understood that ROS production is the key factor for testicular disorders [3]. In this regard, oxidative stress is well documented to be a main contributer in testicular injury which inhibits both spermatogenesis and steroidogenesis [9,10]. Among scholars, the fact that infections are one of the main reasons resulting in oxidative damage in testicular tissues has received great attention [9,10]. Studies have also shown that LPS or infection has short- or long-term impairing effects on male fertility. In this regard, injection of LPS in rats was reported to cause oxidative damage in testicular tissue which led to a lower release of testosterone and disturbance in the spermatogenesis process [3,11]. According to the previous findings, injection of LPS in mice also increased germ cell apoptosis of testicular tissue which finally impaired the spermatogenesis process and motility of sperms [12]. It was also observed that injection of a high dose of LPS caused a rapid reduction of testosterone concentration in mice [13]. Nevertheless, many studies have demonstrated that the administration of LPS to laboratory animals resulted in a transient or permanent detrimental effect on the testicular structure and the spermatogenesis process [[14], [15], [16]].

Olibanum (*Boswellia serrata*) is a member of the Burseraceae family which is also known as Gajabhakshya [17,18]. It is grown in many countries with dry hills such as Northwest India, Eastern Africa, and Arabia [17,19,20]. This plant has been used for medical purposes for thousands of years. The resin collected from this plant is called Frankincense [21], Guggul, incense, or olibanum [19,22,23].

In recent years, the antioxidative or anti-inflammatory effects of *Boswellia serrata* have attracted great attention among researchers [24]. Many studies have also revealed that treatement by *Boswellia serrata* decreases the NF-κB/TNF-α expression and increases the Nrf2/HO-1 expression [[25], [26], [27]]. Moreover, some studies have reported that the administration of *Boswellia serrata* prevents apoptosis via enhancing gene expression Bax/Bcl2 and decreasing the expression of caspase-3 [28]. In addition, many shreds of evidence have shown that *Boswellia serrata* has a beneficial effect on various oxidative stress and inflammatory-related diseases including brain tumors, ulcerative colitis, respiratory inflammatory disorders, and fertility [29]. The anti-inflammatory or antioxidant properties of boswellic acids have also been documented in numerous clinical studies [[30], [31], [32], [33]]. Given the potential balancing properties on oxidative stress and inflammation, this study was designed to determine the effects of olibanum on oxidative stress indicators in testes and sperm parameters in a LPS induced systemic inflammation model in rats.

## 2 Material and methods

### 2.1 Animal groups and treatments

To conduct this study, male Wistar rats (n = 28) weighing 250 ± 10 g were provided by the Central Animal House at School of Medicine. Animals were located in standard animal cages in a room under standard conditons including regulated light and dark cycle (12 h light/12 h dark) and standard environment temperature (22 ± 2 °C) and humidity (55 ± 5 %). The rats had free access to food and water. This experiment was performed following the Guiding Principles in the Care and Use of Animals and the procedures were approved by the local Ethical Commission at Gonabad University of Medical Sciences. The animals were randomly divided into four groups as follows: group 1 or the control group received 1 ml/kg of saline, instead of LPS, and 1ml/kg of 10 % DMSO, instead of olibanum; group 2 or LPS group which received a daily injection of 1 mg/kg LPS [34] and 1ml/kg of 10 % DMSO; group 3 or LPS + Olibanum 100; and group 4 or LPS + Olibanum 200 groups which treated by 100 or 200 mg/kg of olibanum injection besides LPS daily [[35], [36], [37]].

The rats received 14 days of treatments and finally, they were sacrificed. Afterwards, the testes were removed for histological analysis. LPS (*E. coli 055: B5*) was bought from Sigma (Sigma Aldrich Chemical Co.). The chemicals used for othe biochemical tests were obtained from Merck Company. Using a high-performance liquid chromatography method which was done at the Faculty of Medicine, Mashhad University of Medical Sciences, Mashhad, Iran, 3-acetyl-11- keto-b-boswellic acid (AKBA) was reported to be the main component of olibanum extract [38].

### 2.2 TUNEL staining and apoptotic cells quantification

The level of apoptotic cells in testicular tissue was estimated using a TUNEL kit (Roche, Germany). The method was as it was explained in previous studies [39].

The morphometric method was used to determine the number of apoptotic cells in the seminiferous tubules of rats' testis. A 10,000 μm^2^ counting frame was used to count the numbers of apoptotic germ cells per unit area (NA) of testicular tissue and, then, calculated by the below formula:

$$N_{A}=\frac{\sum Q}{a/f.\sum P}$$

in this formula, “ΣQ” indicates the number of enumerated apoptotic cells observed in the sections, “a/f” is the area connected with each frame, and “ΣP” is the sum of frames connected with the points hitting space [40].

### 2.3 Epididymal sperm sampling

The cauda epididymis were dissected and placed in phosphate-buffered saline (PBS, 5 ml) (at 37 °C and 5 % CO2) and the sperms were allowed to swim for 30 min. An aliquot of each sample was used to analyze the sperm count and the morphology was assessed by toluidine blue (TB) staining.

### 2.4 Epididymal high and seminiferous diameter

The tissue sections were provided from the testes. An objective lens ( × 40) of a microscope and a great-resolution camera (dp12) were used and the images were randomly provided. Commercial software was then used to process the images and the epididymal high and seminiferous diameters were measured and calculated [41,42].

### 2.5 Sperm count

Protocols for counting sperms have been demonstrated in previous publications [39]. Briefly, sperm suspension was diluted at the 1: 5 ratio. 10 μl from the sperm suspension was collected into a Neubauer hemocytometer. In the next step, after settling sperms, a light microscope with 400× magnification was used to count the sperm.

### 2.6 Sperm morphology assessment

The method for the evaluation of normal sperm morphology has been explained in previous studies [39]. The Papanicolaou staining was used for evaluating normal sperm morphology. Briefly, sperm smears were washed for 3 min with distilled water. Then, sperm smears for 5 min stained with hematoxylin. In the next step, the sperm smears were washed with distilled water. Afterwards, the smears were soaked in acid alcohol. Next, the sperm smears were washed in running water. Then, for 15 s, they were laid in 96 % alcohols I and II. Then, smears in order were stained with OG6 for 5 min, placed in 96 % alcohol I and II for 15 s, stained with EA 50 for 5 min, and were laid in 96 % alcohols I and II for 15 s and 100 % alcohol for 1 min. In the final step, for each slide, 200 spermatozoa were assessed by a light microscope at the magnification of·1000x. Different forms of sperm morphology count were conducted.

### 2.7 Weight and volume of testis

After sacrificing the animals, the testes were immediately collected and the weight of the testis was precisely measured. To calculate the testis volume, we used the following formula:

$$Vt=\frac{\text{Secondary}\;\text{weight}\_\text{Primary}\;\text{weight}}{\text{The}\;\text{density}\;\text{of}\;\text{the}\;\text{solution}}\times 100$$

secondary weight: (Beaker‏ + Basket‏ + Normal saline ‏ + Testis), Primary weight: (Basket +‏ Normal saline + Beaker‏). The density of the normal saline solution was 1.004.

### 2.8 Biochemical measurements

The methods which were used for evaluation of malondialdehyde (MDA) and total thiol concentration have been eluciated in the previous studies [43,44]. Superoxide dismutase (SOD) activity was estimated by Madesh and Balasubramanian colorimetric assay method [45] and catalase (CAT) activity was estimated by adopting the Aebi method [46].

### 2.9 White blood cell (WBC) count

The blood was collected from the heart and the WBC count was done using an automatic analyzer in a medical laboratory.

### 2.10 Statistical analysis

All the data were provided as means ± SEM and One-way ANOVA followed by Tukey's post-hoc comparison test were used to analyze the data. Differences were considered to be statistically significant at P < 0.05.

## 3 Results

### 3.1 Apoptosis

**Fig. 1:**
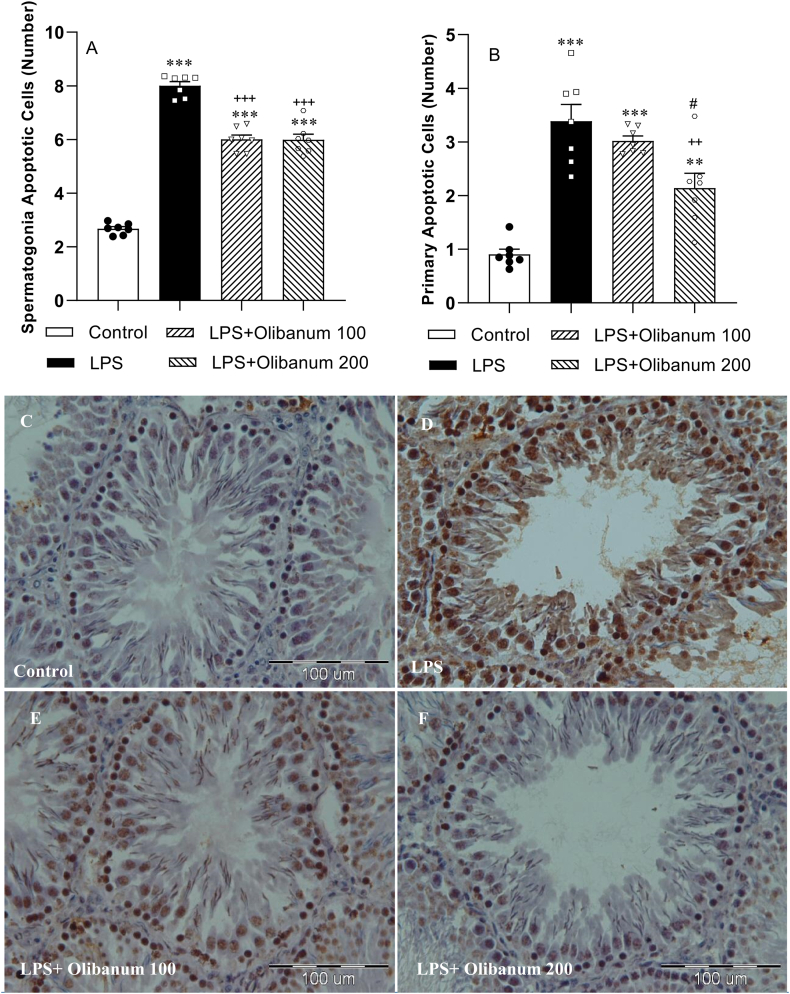
Comparison of TUNEL-positive spermatogonia (A) and primary spermatocytes (B) among the different groups. The data were provided as mean ± SEM. **P < 0.01 and ***P < 0.001 in comparison with the control, ^++^P<0.01 and ^+++^P<0.001 in comparison with the LPS. ^#^P < 0.05 in comparison with LPS + Olibanum 100 group. Parts C–F show apoptotic cells in the photomicrographs of testicular tissues.

Fig. 1 C- 1 F show TUNEL-positive cells in the photomicrographs of testicular tissues.

Spermatogonia and primary apoptotic cells were significantly increased in the LPS group compared to the control rats (both P < 0.001). Spermatogonia apoptotic cells in both LPS + Olibanum 100 and LPS + Olibanum 200 groups (both P < 0.001) and primary apoptotic cells in LPS + Olibanum 200 group (P < 0.01) were significantly decreased in comparison to the LPS group, but no significant difference was obsereved in primary apoptotic cells between LPS + Olibanum 100 and LPS groups. The number of spermatogonia and primary apoptotic cells in both LPS + Olibanum 100 and LPS + Olibanum 200 groups was higher than in the control group (P < 0.01 and P < 0.001). No significant difference was observed between LPS + Olibanum 100 and LPS + Olibanum 200 groups in spermatogonia cells but the primary apoptotic cells in LPS + Olibanum 200 group were lower than in LPS + Olibanum 100 group (P < 0.05; Fig. 1 A and 1 B).

### 3.2 Sperm count and morphology

**Fig. 2:**
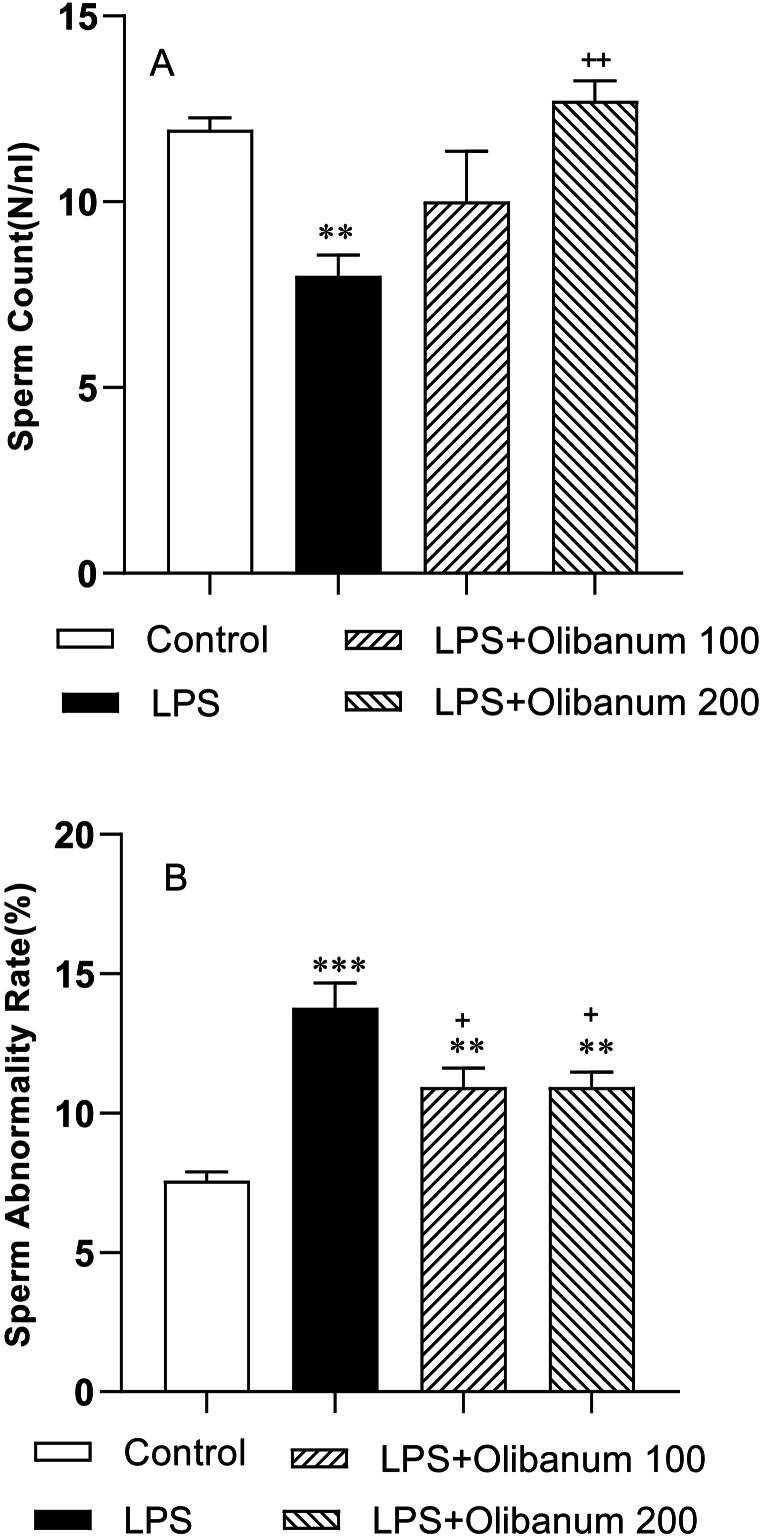
Comparison of sperm count (N/nl) (A), and sperm abnormality (%) (B) in different groups. The data were provided as mean ± SEM. **P < 0.01 and***P < 0.001 in comparison with the control, ^+^P<0.05 and ^++^P<0.01 in comparison with the LPS.

As shown in Fig. 2, the sperm count was decreased while sperm abnormality was significantly increased in the rats of the LPS group compared to those in the control group (P < 0.01 and P < 0.001, respectively) (Fig. 2 A and 2 B). However, sperm count increased significantly in the LPS + Olibanum 200 group in comparison to the LPS group (P < 0.01) (Fig. 2 A) but no difference was seen in sperm count between LPS + Olibanum 100 and LPS groups. Sperm abnormality was decreased in both LPS + Olibanum 100 and LPS + Olibanum 200 groups compared to the LPS group (both at P < 0.05), but they were still higher than in the control group(both P < 0.01). No significant differences were observed between LPS + Olibanum 100 and LPS + Olibanum 200 groups (Fig. 2 B).

### 3.3 Epithelial high and seminiferous diameter

**Fig. 3:**
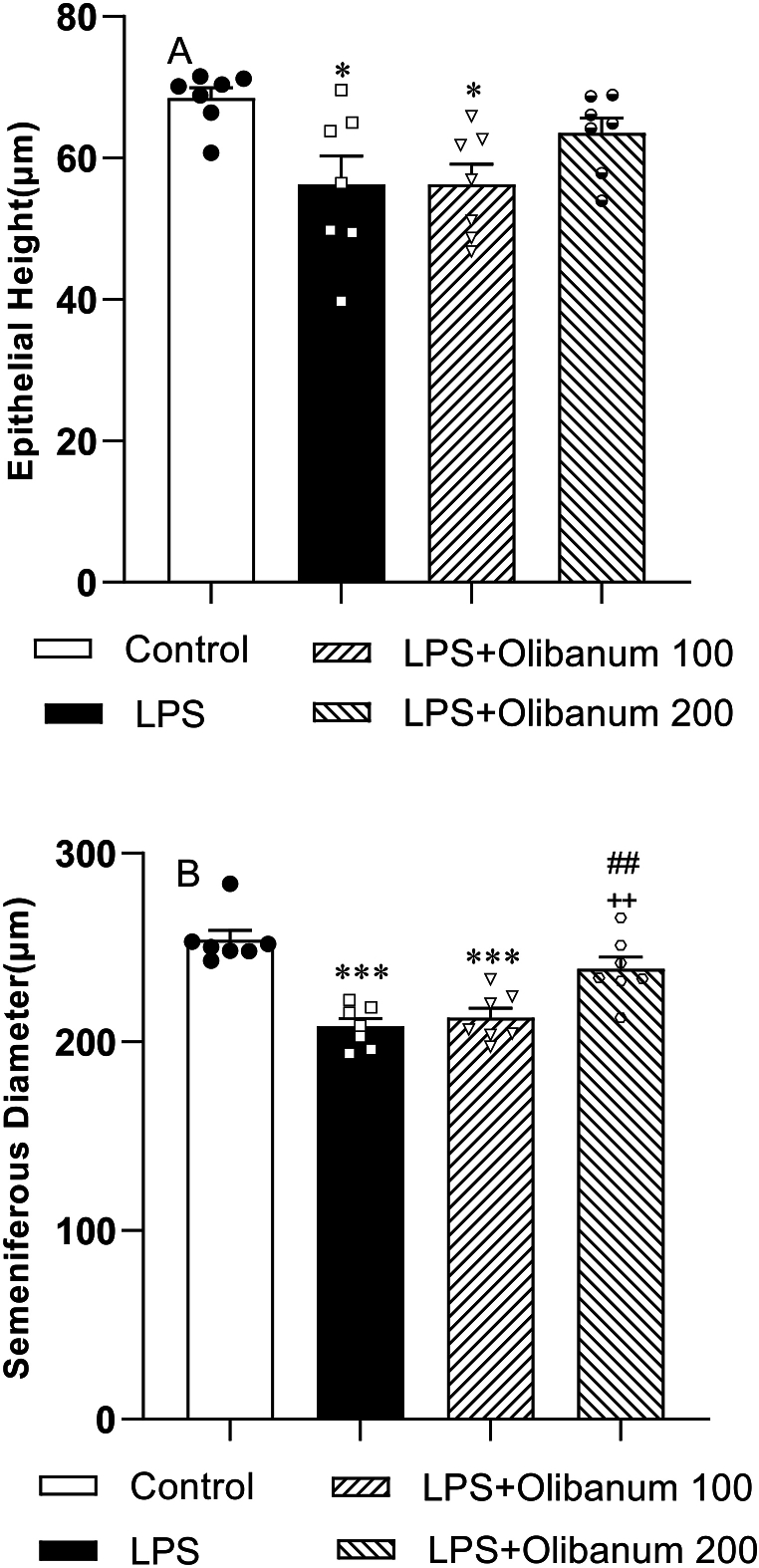
Comparison of epithelial height (A), and seminiferous diameter (B) in different groups. The data were provided as mean ± SEM. *P < 0.05 and ***P < 0.001 in comparison with control, ^++^P<0.01 in comparison with LPS. ^##^P < 0.01 in comparison with LPS + Olibanum 100.

Injection of LPS into the rats revealed a significant reduction in the epithelial height of seminiferous tubules compared to the control group (P < 0.05) (Fig. 3 A). No significant difference was observed in the epithelial high between the LPS + Olibanum 200 or higher the LPS + Olibanum 100 compared to the LPS group. In the LPS + Olibanum 100 group, the epithelial height of seminiferous tubules was lower than the control group (P < 0.01) but there was no significant difference between the LPS + Olibanum 100 and control groups (Fig. 3 A). Seminiferous diameter in the LPS group was significantly lower than in the control group (P < 0.001) however, the high dose of olibanum (200 mg/kg) significantly increased seminiferous diameter compared to the LPS + Olibanum 100 and LPS groups (P < 0.01 for both). In both LPS + Olibanum 200 group, the seminiferous diameter was lower than that of the control group (P < 0.001) but no difference was seen between LPS + Olibanum 100 and the control groups (Fig. 3 B).

### 3.4 Testis weight and volumeur

**Fig. 4:**
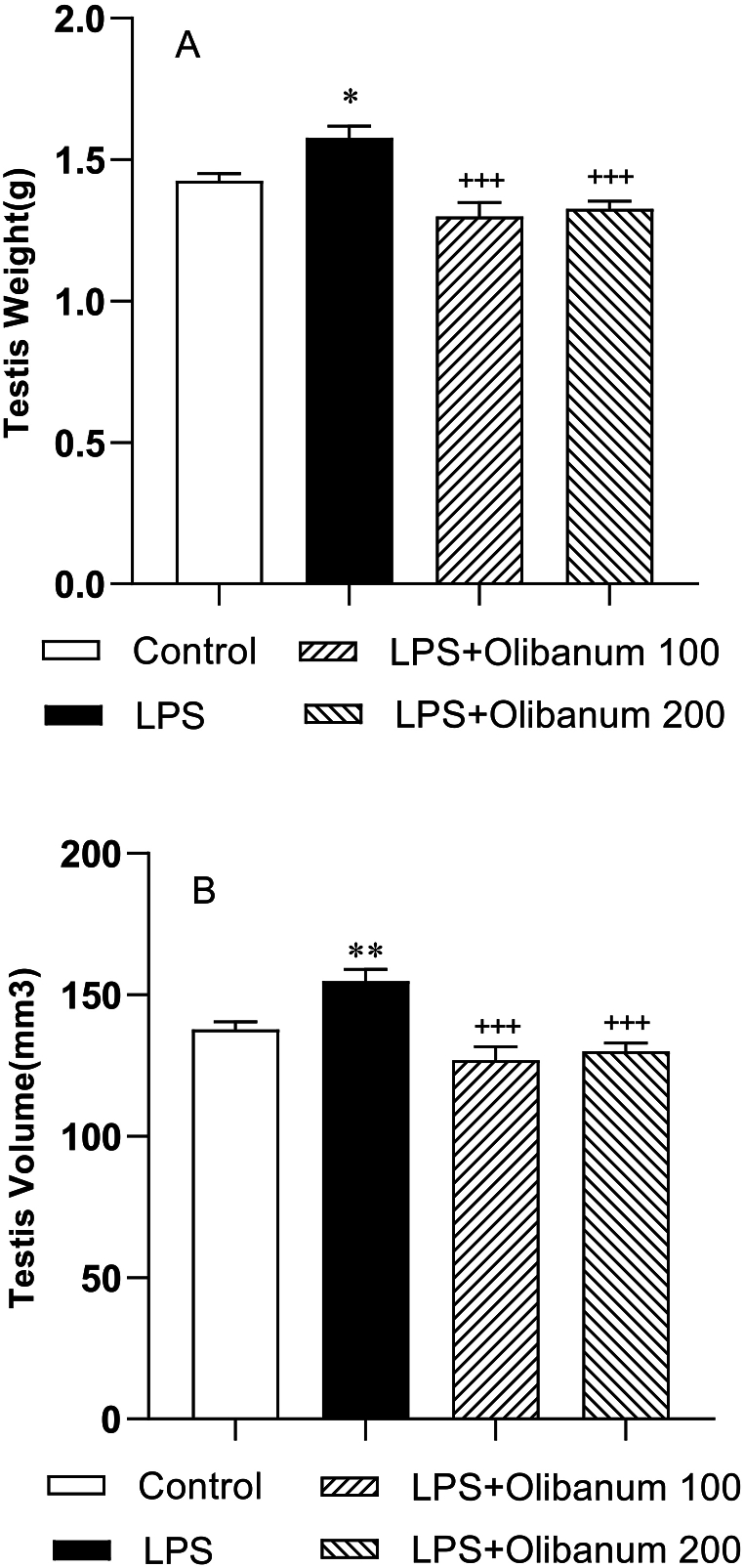
Comparison of testes weight (A), and volume (B) in different groups. The data were expressed as mean ± SEM. *P < 0.05 and **P < 0.01 in comparison with control, ^+++^P<0.001 in comparison with LPS.

In the LPS group, both testis weight (Fig. 4 A) and testis volume (Fig. 4 B) were higher than in the control group (P < 0.05 and P < 0.01 respectively). Administering of olibanum significantly reduced both testis weight (Fig. 4 A) and volume (Fig. 4 B) in both LPS + Olibanum 100 and LPS + Olibanum 200 groups compared to the LPS group (P < 0.001 for both).

### 3.5 Results of the biochemical assessment

**Fig. 5:**
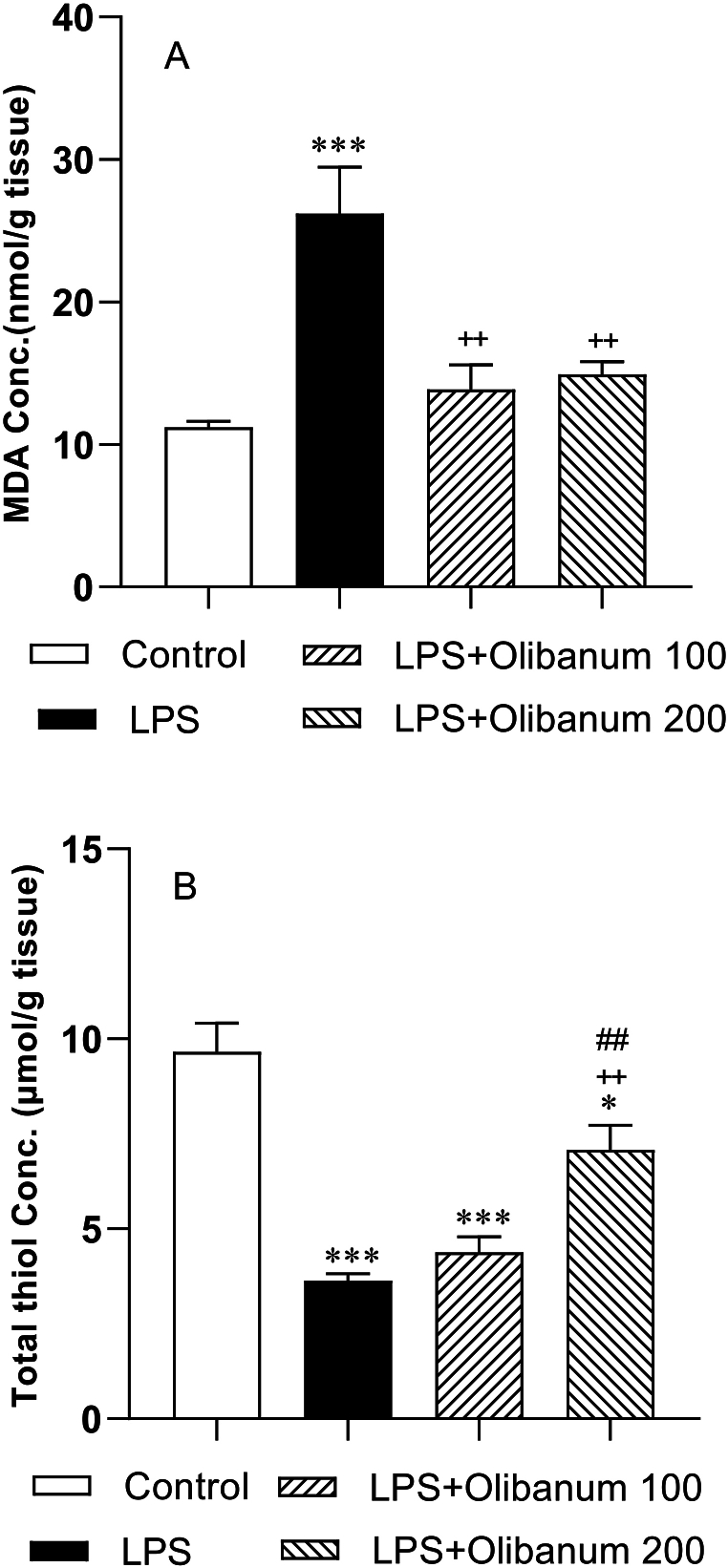
Comparison of MDA (A), and thiol (B) in the testes of different groups. The data were expressed as mean ± SEM. *P < 0.05 and ***P < 0.001 in comparison with control, ^++^P<0.01 in comparison with LPS.

Significantly more MDA was detected in the testes of the LPS group compared to the control group (P < 0.001). Both doses of olibanum (100 and 200 mg/kg) had a reducer effect on the concentrations of MDA in the testicular tissue of LPS + Olibanum 100 and LPS + Olibanum 200 groups compared with the LPS group (P < 0.01) (Fig. 5 A).

As a result of LPS treatment, thiol concentrations in the testes of the LPS group were decreased in comparison with those in the control group (P < 0.001). Administering the high dose Olibanum (200 mg/kg) revealed a significant increase in the thiol concentrations of testes compared to the LPS and LPS + Olibanum 100 groups (both P < 0.01). The concentrations of thiols of testes tissue in the LPS + Olibanum 100 and LPS + Olibanum 200 groups were lower than in the control group (P < 0.001 and P < 0.05 respectively). However, the difference in the thiol concentrations between the low dose of olibanum (100 mg/kg) and the LPS group was not significant (Fig. 5 B).

**Fig. 6:**
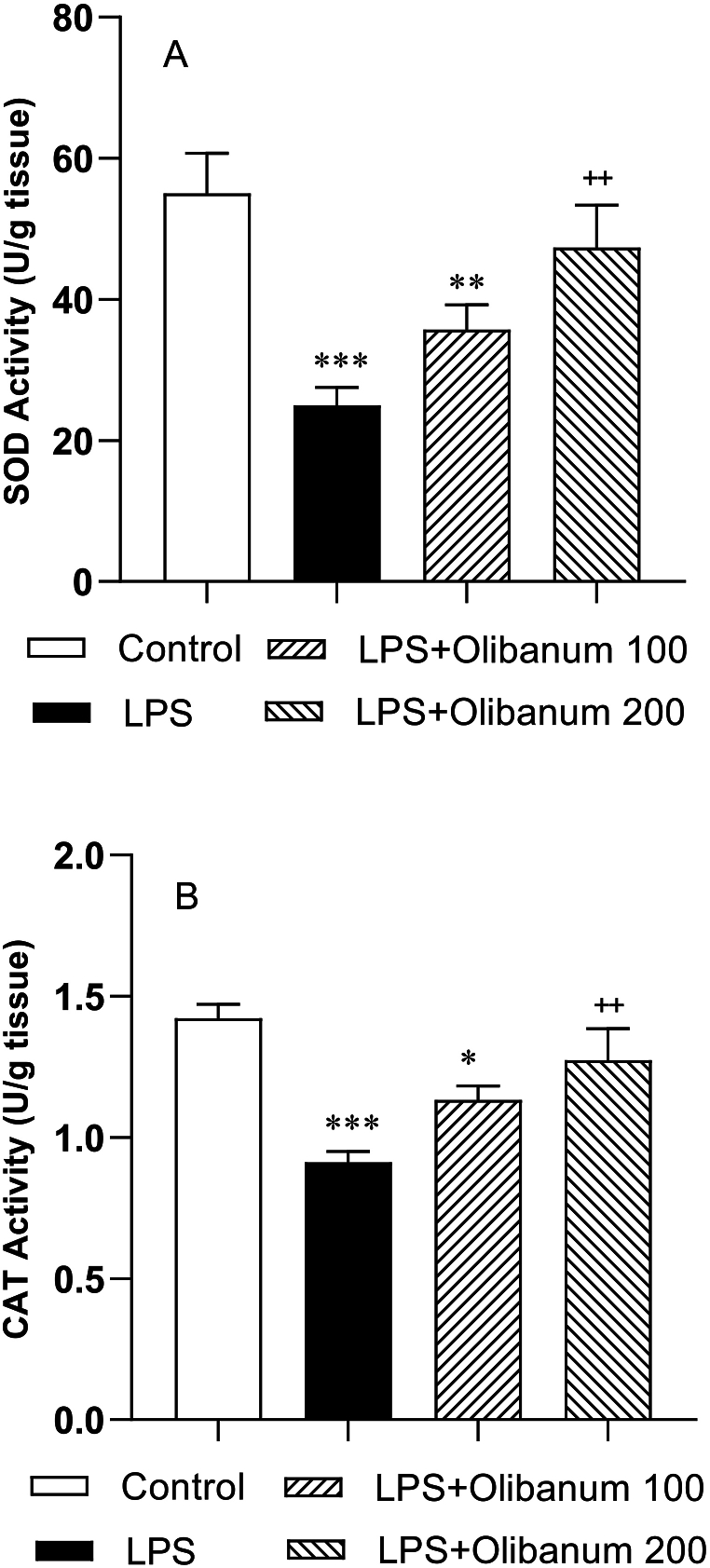
Comparison of SOD (A), and CAT (B) in the testes of different groups. The data were expressed as mean ± SEM. *P < 0.05, **P < 0.01 and ***P < 0.001 in comparison with control, ^++^P<0.01 in comparison with LPS.

A decrease in SOD and CAT activity in the testes of the LPS group was observed in comparison with the control group (P < 0.001 for both). A high dose of olibanum (200 mg/kg) significantly increased the activities of SOD and CAT enzymes in comparison with the LPS group (both P < 0.01) however, the activities of SOD and CAT in testes did not differ significantly between LPS + Olibanum 100 and the LPS groups (Fig. 6 A and 6 B).

### 3.6 Results of the WBC count

**Fig. 7:**
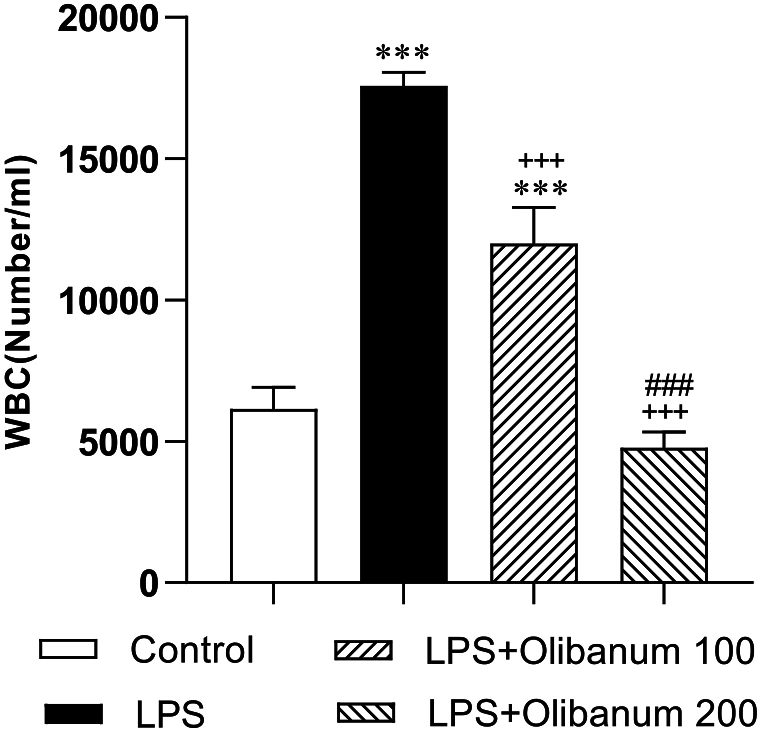
Comparison of the white blood cell (WBC) count in the blood of different groups. Data was expressed as Mean ± SEM. ***P < 0.001 compared to the control, ^+++^P<0.001 compared to the LPS, ^###^P < 0.001 compared to the LPS + Olibanum 100.

The results showed that LPS injection led to an increased count of WBC compared with the control group (P < 0.001). Both 100 and 200 mg/kg doses of the extract decreased WBC count(both P < 0.001). The effect of 200 mg/kg of the extract on WBC count was more effective than 100 mg/kg (P < 0.001). There was no significant difference between LPS + Olibanum 200 and the control group but in the blood of LPS + Olibanum 100 group, the WBC count was still higher than that of the control group (P < 0.001) (Fig. 7).

## 4 Discussion

The results revealed that olibanum had antioxidant effects on testicular tissue and revealed some beneficial effects on testicular tissues and sperm parameters damage following LPS injection in rats.

In the present study, daily injection of 1 mg/kg of LPS for 14 days led to an increased count of WBC, which confirmed a systemic inflammation condition. Injection of LPS was also led to a decrease in the activities of antioxidant enzymes including CAT and SOD, and consntration of thiol while enhancing the MDA level in the testicular tissues, which confirms an oxidative stress condition in testes of the rats. In addition, LPS injection reduced the sperm count and normal morphology while increasing the number of apoptotic cells of spermatogonia, and primary cells in testicular tissue. These results are in agreement with those studies, in which LPS administration to rats led to a damage to the spermatogenesis process and testicular structure by inducing oxidative stress and apoptosis [3,8,47,48]. Using laboratory animals, it is suggested that the failure of testicular cells to eliminate H2O2 is due to a decline in CAT and/or SOD activity [49]. In addition, an overproduction of H2O2 reduces glutathione-s-transferase (GST) activity [50] and decreases glutathione (GSH) levels [51]. All of these events led to oxidative stress damages to both Leydig cell steroidogenesis and spermatogenesis and are considered to be the main reason for testicular dysfunction induced by LPS or infection [9,10]. Oxidative stress conditions also decreases the gene expressions of steroidogenic enzymes such as 3β-hydroxysteroid dehydrogenase (3 β -HSD), 17 β -hydroxysteroid dehydrogenase (17 β -HSD), and a repress in androgen receptor corepressor-19 kDa (ARR19) in testicular tissue [4]. Reddy et al. (2006) also reported that systemic inflammation due to LPS injection in adult rats increased 4-hydroxy-2-nominal (HNE) as a special apoptotic inducer and reduced steroidogenic acute regulatory (StAR) protein in testicular tissue, which finally caused damage to spermatogenesis [3].

Overproduction of cytokines after LPS injection activates macrophages which finally causes ROS generation [52]. Spermatogonia and spermatozoa are vulnerable to ROS attack [53]. According to this evidence, it is suggested that a part of injuries in testes from the injection of LPS in our study is due to an increase in ROS generation and high susceptibility of germ cells. In addition, the produced ROS in the testes of rats injected with LPS can impair the mitochondrial electrochemical gradient which may reduce proteolytic processing of StAR; finally, these events lead to the reduction of cholesterol preparation to testosterone production. Thus, administration of LPS may be through an increase in ROS production leading to disrupting the mitochondrial electrochemical gradient, then causing damage to Leydig cells, and finally reducing the production of testosterone [11,54]. Furthermore, it has also been reported that treating adult rats with LPS can result in a decrease in serum and intratesticular testosterone concentration through direct impairment in the steroidogenesis process of Leydig cells [16]. Furthermore, the cytoplasmic membrane of germ cells contains a high concentration of polyunsaturated fatty acids which makes them more sensitive to oxidative stress [55].

Our results also showed that injection of LPS for 14 days in rats led to spermatids and spermatogonia apoptosis. In confirmation of our results, it was previously shown that treating rats with LPS caused epithelial disorganization, apoptosis in seminiferous epithelium cells, degeneration of the early round spermatids, and apoptosis of early pachytene spermatocytes [16]. Kajihara et al. concluded in their report that apoptosis induced by LPS in germ cells is due to the direct effect of LPS on spermatogonia [12]. The main LPS receptor found in sperm and the epididymal epithelium is toll-like receptor 4 (TLR4) [56,57]. The TLR4 signaling pathway is activated by LPS and, then, it activates the nuclear factor kappa light chain enhancer of activated B cells (NFκB), which moves into the nucleus. In addition, LPS regulates the transcription of TLR4-activated cytokines including tumor necrosis factor α (TNFα) [58]. On the other hand, oxidative stress status or inflammatory responses induced by LPS can result in apoptosis in testicular tissue [47]. Therefore, the apoptosis that was seen in the present study is suggested to be due to the direct effect of LPS or its indirect effect through inducing oxidative stress status and/or inflammatory response.

Cosidering the adverse effects of oxidative stress, more attention has been drawn to the beneficial therapeutic properties of antioxidants. In previous studies, it was shown that the anti-oxidant agents protected the function and structure of testes [39,59]. Using DPPH and FRAP methods, olibanum has been observed to have antioxidant properties [60]. Also in animal studies, olibanum has shown anti-oxidant effects [37]. Our results revealed that treatment by olibanum for 14 days improved the antioxidant markers including thiol level, as well as the activities of CAT, and, SOD while decreasing MDA concentration in the testicular tissues. The current results also represented that the extract in both doses reversed WBC count which confirms its anti-inflammatory effects. The results also showed that spermatogonia apoptotic cells in both LPS + Olibanum 100 and LPS + Olibanum 200 groups and primary apoptotic cells in LPS + Olibanum 200 group were lower than those in the LPS group. In addition, olibanum at 200 mg/kg improved sperm count and seminiferous diameter, and both 100 and 200 mg/kg of olibanum decreased sperm abnormality rate. Inconsistent in our results, Nusier et al. (2007) observed that oral administration of olibanum in rats increased the average weights of the epididymis, ventral prostate, and seminal vesicles. They also reported that olibanum enhanced sperm density and sperm motility in testes of rats [61]. Collectively, the results of the current research revealed that olibanum attenuated LPS-induced testicular damage, apoptosis, and oxidative stress. However, more studies are suggested to be done using more precise methods including PCR and western blotting.

In the current study, the compounds responsible for the beneficial effects of olibanum on testicular tissue was not evaluated and it needs to be investigated in the future. Olibanum is reported to be containing triterpenes inclusing *α*- and *β*-boswellic acids, lupeolic acid (30–60 %) essential oils (5–10 %), and polysaccharides (20–35 %) [60,62]. Tannins, flavonoids, terpenoids, carbohydrates, alkaloids, phenolic compounds, phytosterols, saponins, proteins, and glycosides are also reported to be present in the olibanum extract [60]. Using a high-performance liquid chromatography method, 3-acetyl-11- keto-b-boswellic acid (AKBA) was previously reported to be the main component of olibanum extract [38]. In addition, AKBA is reported to be responsible for the antioxidant and anti-inflammatory properties of olibanum [38,63]. We also previously reported that olibanum attenuated neuroinflammation and oxidative stress in the brain [37,64], which was related to AKBA [36,65]. Previous studies have reported that boswellic acid has efficient antioxidant activity in different organs including the brain, kidneys, testes, and cardiovascular systems [37,66,67]. In the previous studies, oral administration of boswellic acid as a main ingredient of olibanum increased spermatogenesis, which was due to its antioxidant activity [66,68]. It was also previously shown that oral treatment by boswellic acid in male rats enhanced GSH and testosterone and decreased MDA, TNF-α, and interleukin-6 levels [66]. They demonstrated that boswellic acid upregulated gene expression of anti-apoptotic proteins including Bcl-2 and downregulated apoptotic proteins such as heat shock protein, and Bax. Moreover, Sami et al. (2019) demonstrated that boswellic acid upregulated the expression of Bcl2 and downregulated caspase-3 in renal tissue. These anti-apoptotic effects were achieved by increasing antioxidant defense such as glutathione and decreasing oxidant markers such as MDA [69]. In the same line, Ahmed et al. (2020) confirmed the protective effect of boswellic acid on testicular tissue damage induced by testicular torsion/detorsion. They demonstrated that the protective effects of boswellic acid were via a decreased level of oxidative stress criteria or increased level of antioxidant markers which finally reduced apoptosis or improved spermatogenesis [70]. Considering this evidence, AKBA seems to be responsible for the benefits of olibanum which was obsereved in the current research; however, it needs to be precisely investigated in future studies.

## 5 Conclusion

In the current research, systemic inflammation, oxidative stress, abnormal sperm parameters, and testicular apoptosis were seen after LPS injection. Olibanum administration was able to reveal some beneficial effects against inflammation related testicular damage. Thus, olibanum may be useful for attenuating the complications resulting from inflammation.

## Compliance with ethical standards

All applicable international, national, and institutional guidelines for the care and use of animals were followed. All the experimental protocols and procedures were approved by the Ethics Committee of Gonabad University of Medical Sciences (IR.GMU.REC.1398.002).

## Data availability statement

The datasets are available from the corresponding author upon reasonable request.

## CRediT authorship contribution statement

**Narjes Jalilvand:** Visualization, Investigation, Data curation. **Yousef Baghcheghi:** Writing – original draft, Formal analysis, Data curation. **Masoumeh Fani:** Visualization, Formal analysis, Data curation. **Farimah Beheshti:** Methodology, Investigation, Formal analysis, Data curation. **Alireza Ebrahimzadeh-Bideskan:** Writing – review & editing, Supervision, Software, Formal analysis, Conceptualization. **Narges Marefati:** Visualization, Methodology, Formal analysis, Data curation. **Maryam Moghimian:** Writing – review & editing, Supervision, Funding acquisition. **Mahmoud Hosseini:** Writing – review & editing, Supervision, Conceptualization.

## Declaration of competing interest

The authors declare that they have no known competing financial interests or personal relationships that could have appeared to influence the work reported in this paper.
